# Are older adults using ChatGPT for medical advice? Results from a cross-sectional survey study

**DOI:** 10.64898/2026.01.01.25342468

**Published:** 2026-01-04

**Authors:** Meghan Reading Turchioe, Afra Shamnath, Jarrott Mayfield, Orson Xu, David Slotwiner, Natalie Benda

**Affiliations:** 1 Columbia University School of Nursing, New York, NY, USA; 2 Johns Hopkins University School of Public Health, Baltimore, MD, USA; 3 Columbia University Department of Biomedical Informatics, New York, NY, USA; 4 Weill Cornell Medicine Department of Population Health Sciences, New York, NY, USA

## Abstract

General-purpose large language models (LLMs) like ChatGPT are increasingly used for medical advice despite lacking medical training and frequently producing incorrect or unsafe output. Older adults’ health information-seeking behaviors using LLMs remain poorly characterized. We conducted a cross-sectional survey of 574 US adults aged 50+ recruited via Prolific, balanced by sex and race. Participants reported health information sources, ChatGPT and PubMed use, demographics, and health literacy. Most participants (92%) searched online for health information. All had heard of ChatGPT, and 63% used it for medical information, compared to 44% who had heard of PubMed and 39% who used it. Those with inadequate health literacy had higher odds of ChatGPT use for medical advice (AOR 2.36, 95% CI 1.30–4.52) versus those with adequate health literacy. In conclusion, with more than half of older adults using LLMs for medical advice, the development of safer, purpose-trained medical LLMs is warranted.

## Introduction

Health information-seeking behaviors, referring to ways of gathering health-related information, are associated with the ability to self-manage health and, ultimately, health outcomes.^1^ Adults in the U.S. may use general-purpose large language models (LLMs), such as ChatGPT, for medical advice.^2^ LLMs provide natural language responses to user queries that are easy to understand, but are not trained or tested for medical purposes. One review found 88% of included studies evaluating LLMs for medical purposes reported incorrect or incomplete output, and nearly half reported unsafe output.^3^ Chronic conditions affect 80–90% of adults age 35 and older,^4^ creating substantial medical information needs. However, older adults’ health information-seeking behaviors in the new era of broadly accessible LLMs have not been well characterized. This study investigated health information-seeking behaviors, including LLM use, among US adults aged 50 and older.

## Methods

This cross-sectional survey study used Prolific to recruit participants balanced by sex and reflecting 2020 US Census racial distribution (58% White, 14% Black, 6% Asian, 20% other, and 2% mixed). Eligible participants could read English and were aged 50 and older.

Survey items were adapted from the Health Information National Trends Survey and a separate survey assessing online health information search habits and sources.^5^ We asked about familiarity with and frequency of using ChatGPT (selected because it is the most commonly used LLM with over 700 million users as of September 2025). To contrast this, we also asked about their use of PubMed (a federally supported biomedical library). Participants self-reported demographics and health literacy, completing surveys via Qualtrics. Quality was audited through attention checks and response times. Participants completing the questionnaire and passing attention checks received $16 per hour. The Columbia University Institutional Review Board approved this study.

We used descriptive statistics and logistic regression to examine demographic effects on ChatGPT use for health information (binarized as never versus any use). Analyses were performed using R version 4.1.2.

## Results

Of 622 participants recruited, 48 were excluded (33 age ineligible, 9 incomplete surveys, 6 failed attention checks; 0 excluded for low response times), yielding 574 participants. Mean age was 58 (SD 6.8) years; 51% were female, 70% White, 16% Black or African American, and 10% Hispanic or Latino. Education levels were 30% high school or less, 38% Bachelor’s degree, and 32% graduate degree; 71% had adequate health literacy. Most (93%) used the Internet more than once daily.

Most participants (92%) used the Internet to search for health information (Table 1). All but two had heard of ChatGPT (100%), and 63% used it at least once for medical information. Fewer had heard of PubMed (44%) or used it (39%). Other common sources included licensed healthcare professionals (93%) and medical websites (75%). Fewer used government health agencies (40%), news websites (16%), and social media (15%).

In adjusted models (Table 2), Asian participants had lower odds of using ChatGPT for medical information versus White participants (adjusted odds ratio [AOR] 0.36, 95% confidence interval [CI] 0.16–0.78). Participants with graduate degrees (AOR 2.57, 95% CI 1.51–4.43) and Bachelor’s degrees (AOR 1.66, 95% CI 1.01–2.72) had higher odds of using ChatGPT versus those with high school education or less. Those with inadequate health literacy had higher odds of ChatGPT use (AOR 2.36, 95% CI 1.30–4.52) versus those with adequate health literacy.

## Discussion

Over 60% of adults aged 50 and older have used ChatGPT for medical information, with use varying by race, education, and health literacy. By contrast, our recent Prolific study of younger participants (mean age 39) reported lower rates (33%) of ChatGPT use for medical information.^6^ Findings align with our prior work showing that adults with lower healthy literacy are more open to using AI-based tools in their care.^7^ A limitation of this study includes that we recruited participants via an online sample, who may have been predisposed to using AI-based tools and may differ from other adults aged 50 and older. Future work should develop purpose-trained medical LLMs that prioritize safety and usability for older adults with low health literacy.

## Figures and Tables

**Table 1.** Health information seeking among survey participants (n=574)

**Tried looking for information about your health from any source**	553 (96.3%)
**Used the Internet for these health-related needs in the last 12 months**	
Look for health or medical information	525 (91.5%)
View medical test results	349 (60.8%)
Made an appointment with a health care provider	331 (57.7%)
Send a message to a healthcare provider	325 (56.6%)
**Heard of ChatGPT**	572 (100%)
**If yes, frequency of ChatGPT use for medical information**	
Often	99 (17.2%)
Sometimes	154 (26.8%)
Once or twice	111 (19.3%)
Never	208 (36.%)
N/A- have not used ChatGPT	2 (0.3%)
**Heard of the online biomedical library, PubMed**	254 (44.3%)
**If yes, frequency of PubMed use for medical information**	
Often	27 (4.7%)
Sometimes	116 (20.2%)
Once or twice	81 (14.1%)
Never	30 (5.2%)
N/A- have not used PubMed	320 (55.7%)
**Sources relied upon for information about personal health**	
A licensed healthcare professional (i.e., doctor or nurse)	531 (92.5%)
Medical website (i.e., WebMD)	428 (74.6%)
Scientific researchers	336 (58.5%)
Government health agencies	232 (40.4%)
Other people with the same health condition	193 (33.6%)
Family, friend, or community member	177 (30.8%)
News website	93 (16.2%)
Social media (i.e., Facebook or Instagram)	87 (15.2%)
Religious organizations and leaders	15 (2.6%)

**Table 2:** Differences in frequency of ChatGPT use for medical advice by sociodemographic characteristics, n=574

	Never (n=208)	Any use (n=364)	Unadjusted OR (95% CI)	p value	Adjusted OR (95% CI)	p value
**Age**						
Reference: 50–59	122 (33%)	246 (67%)	—	—	—	—
60–69	67 (41%)	95 (59%)	0.70 (0.48–1.03)	0.070	0.76 (0.48–1.21)	0.247
70+	19 (45%)	23 (55%)	0.60 (0.32–1.15)	0.121	0.76 (0.36–1.63)	0.476
**Sex**						
Reference: Female	113 (39%)	179 (61%)	—	—	—	—
Male	95 (34%)	185 (66%)	1.23 (0.87–1.73)	0.236	1.04 (0.69–1.57)	0.845
**Race**						
Reference: White	148 (37%)	254 (63%)	—	—	—	—
Black or African American	19 (21%)	72 (79%)	2.21 (1.30–3.90)	0.004	1.83 (0.98–3.55)	0.063
Asian	20 (53%)	18 (47%)	0.52 (0.27–1.02)	0.058	0.36 (0.16–0.78)	0.010
American Indian or Alaska Native	7 (47%)	8 (53%)	0.67 (0.23–1.93)	0.441	0.53 (0.15–1.83)	0.316
Native Hawaiian or Other Pacific Islander*	0 (0%)	1 (100%)	—	—	—	—
Prefer not to answer*	14 (56%)	11 (44%)	—	—	—	—
**Ethnicity**						
Reference: not Hispanic/Latino	191 (38%)	314 (62%)	—	—	—	—
Hispanic/Latino	16 (29%)	40 (71%)	1.52 (0.84–2.86)	0.176	1.67 (0.78–3.86)	0.206
Prefer not to answer*	1 (9%)	10 (91%)	—	—	—	—
**Education**						
Reference: High school or less	85 (50%)	86 (50%)	—	—	—	—
Bachelor’s degree	78 (36%)	139 (64%)	1.76 (1.17–2.65)	0.007	1.66 (1.01–2.72)	0.045
Graduate degree (Master’s or higher)	44 (24%)	139 (76%)	3.12 (2.00–4.94)	<0.001	2.57 (1.51–4.43)	<0.001
Prefer not to answer*	1 (100%)	0 (0%)	—	—	—	—
**Finances**						
Reference: More than enough	22 (39%)	35 (61%)	—	—	—	—
Enough	95 (28%)	245 (72%)	1.62 (0.89–2.89)	0.105	1.78 (0.92–3.43)	0.086
Not enough	83 (52%)	77 (48%)	0.58 (0.31–1.07)	0.087	0.65 (0.31–1.34)	0.244
Prefer not to answer*	8 (53%)	7 (47%)	—	—	—	—
**Health Literacy**						
Reference: Adequate	185 (40%)	275 (60%)	—	—	—	—
Inadequate	23 (21%)	89 (79%)	3.28 (1.93–5.89)	<0.001	2.36 (1.30–4.52)	0.007
**Health insurance coverage in the last year**						
Reference: Yes, for full year	185 (39%)	294 (61%)	—	—	—	—
No/ Part of the year	21 (24%)	65 (76%)	1.95 (1.17–3.36)	0.013	2.03 (0.93–4.87)	0.091
Prefer not to answer*	2 (29%)	5 (71%)	—	—	—	—
**Type of health insurance of in the past year**						
Reference: Medicaid	58 (34.3%)	111 (65.6%)	—	—	—	—
Other than Medicaid	150 (37.2%)	253 (62.8%)	0.87 (0.59–1.27)	0.483	0.73 (0.46–1.17)	0.198

* Models did not converge due to too few observations.

## Data Availability

Data, statistical code, and the informed consent document will be made available upon reasonable request after completing a data use agreement.
